# Cognitive Decline and Dementia in the Oldest-Old

**DOI:** 10.5041/RMMJ.10092

**Published:** 2012-10-31

**Authors:** Efrat Kravitz, James Schmeidler, Michal Schnaider Beeri

**Affiliations:** 1 The Joseph Sagol Neuroscience Center, Sheba Medical Center, Ramat Gan, Israel;; 2 Parkinson’s Disease and Movement Disorders Clinic, Department of Neurology, Sheba Medical Center, Ramat Gan, Israel; and; 3 Department of Psychiatry, Mount Sinai School of Medicine, New York, NY, USA

**Keywords:** Dementia, epidemiology, neurobiology, oldest-old, risk factors

## Abstract

The oldest-old are the fastest growing segment of the Western population. Over half of the oldest-old will have dementia, but the etiology is yet unknown. Age is the only risk factor consistently associated with dementia in the oldest-old. Many of the risk and protective factors for dementia in the young elderly, such as ApoE genotype, physical activity, and healthy lifestyle, are not relevant for the oldest-old. Neuropathology is abundant in the oldest-old brains, but specific pathologies of Alzheimer’s disease (AD) or vascular dementia are not necessarily correlated with cognition, as in younger persons. It has been suggested that accumulation of both AD-like and vascular pathologies, loss of synaptic proteins, and neuronal loss contribute to the cognitive decline observed in the oldest-old. Several characteristics of the oldest-old may confound the diagnosis of dementia in this age group. A gradual age-related cognitive decline, particularly in executive function and mental speed, is evident even in non-demented oldest-old. Hearing and vision losses, which are also prevalent in the oldest-old and found in some cases to precede/predict cognitive decline, may mechanically interfere in neuropsychological evaluations. Difficulties in carrying out everyday activities, observed in the majority of the oldest-old, may be the result of motor or physical dysfunction and of neurodegenerative processes. The oldest-old appear to be a select population, who escapes major illnesses or delays their onset and duration toward the end of life. Dementia in the oldest-old may be manifested when a substantial amount of pathology is accumulated, or with a composition of a variety of pathologies. Investigating the clinical and pathological features of dementia in the oldest-old is of great importance in order to develop therapeutic strategies and to provide the most elderly of our population with good quality of life.

## INTRODUCTION

As life expectancy is steadily increasing,^1^ the Western population is aging. With the decline in fertility, the extreme elderly are the fastest growing segment of the population. In the US alone, the proportion of those aged ≥85 is expected to increase from less than 2% in 2010, to over 4% in 2050, constituting more than 20% of those aged ≥65.^2^ Combining the world’s more developed regions (Europe, Northern America, Australia/New Zealand, and Japan), by the middle of this century 5.5% of the population will be aged ≥85.^3^ The fast increase in the proportion of the oldest-old in the population will impose new public health and economic challenges. Within this age group, over half will have dementia,^4^,^5^ and the annual incidence rate will double every 5 years.^6^ Over 10% of the oldest-old will live in skilled-nursing facilities,^7^ and even more will utilize assisted-living facilities. About 50% of the residents of skilled-nursing facilities in the US are oldest-old.^7^ Middle-aged individuals will find themselves going from caring for their children to caring for their parents. To date, the current knowledge base of the epidemiology, neuropsychology, and neurobiology of dementia in the oldest-old is inadequate for developing therapeutic strategies. Understanding dementia in extremely old age is therefore crucial for easing the economic and societal burden of caring for our most elderly, which will increase dramatically in the next few decades.

Here we review the neuropsychological and neurobiological characteristics of dementia in very old age, and give special attention to risk and protective factors.

## EPIDEMIOLOGY OF DEMENTIA IN THE OLDEST-OLD

Normal aging does not imply unavoidably cognitive decline, and dementia is not an inevitable consequence of old age. According to the DSM-IV, dementia is characterized by the development of multiple cognitive deficits that include memory impairment and at least one of the following cognitive disturbances: aphasia, apraxia, agnosia, or a disturbance in executive functioning; the cognitive deficits must be sufficiently severe to cause impairment in occupational or social functioning and must represent a decline from a previously higher level of functioning.^8^ Estimates of cognitively intact centenarians are 11%^9^ to 30%.^10^–^13^ Among the oldest-old, estimates of dementia prevalence are about 50%^14^ to over 60%.^4^,^5^ Nevertheless, the dementia incidence rate is a matter of controversy. Slowing of dementia incidence after age 90 has been found in several studies,^15^–^21^ but results from the pioneering “90+ Study,” a study of the neuropsychology and neurobiology of over 1,200 nonagenarians, suggest that the incidence of dementia continues to rise exponentially after the age of 90.^22^ The all-cause dementia incidence rate was found to increase from 12.7% per year for those aged 90 to 94 years, through 21.2% per year for the group 95 to 99 years old, to 40.7% per year for persons aged 100 years and older, essentially doubling every 5.5 years.^22^ This increase in incidence rate is comparable with that observed for persons aged 65 to 90, which also doubles approximately every 5 years.^23^ Recent results from the 90+ Study highlights the relevance of the baseline cognitive status of the oldest-old for the observed incidence rate. This study reported that all-cause dementia incidence was highest for participants who, at the beginning of the study, were not demented but had amnestic mild cognitive impairment (MCI) (31.4% per year) and other cognitive impairment (39.9% per year). Participants with normal cognition at the beginning of the study had an incidence of 8.4% per year.^24^ Differences in evaluation methods and attention to baseline cognitive status may account for some of the differences in results between studies.

The most common subtypes of dementia are Alzheimer’s disease (AD) and vascular dementia (VaD). If incidence rates of AD differ from those of VaD, differences in the composition of the cohort, in terms of dementia subtypes, may account for some differences between studies as well. It is therefore interesting to examine whether the incidence rate of each of these dementia subtypes is similar to that of all-cause dementia. Some studies suggested that there are no significant differences in incidence rates between AD, VaD, and all-cause dementia in the oldest-old.^19^,^20^,^25^,^26^ Other studies, however, reported higher incidence rates for AD, which continued to increase with age, as compared to VaD, which remained lower^27^ and fairly stable across age.^28^ The reason for this discrepancy is unclear. One possibility is the dying-off of the individuals who are predisposed to VaD. Those individuals are likely to be survivors of cardiovascular diseases and stroke, and therefore are less likely to reach extremely old age. In addition, the proportion of men and women who suffer from AD is different from this proportion in VaD. With women at greater risk for AD (discussed below) and having a longer life expectancy than men, differences in incidence rates between AD and VaD may be more robust in specific subgroups.

Noteworthy is the fact that diagnosis of dementia in these studies was mostly based on neuropsychological examination, and classifying subtypes of dementia antemortem in the oldest-old is tricky, at best. Furthermore, oldest-old individuals are more likely to suffer from medical comorbidities and have high rates of sensory loss, psychoactive medication usage, frailty, and fatigue (for review see^29^). Together with the decline in cognition often observed in normal aging (discussed below), these factors impose a challenge on the diagnosis of dementia in this unique population, possibly contributing to the variability in incidence rates reported in different studies.

## ETIOLOGY OF DEMENTIA IN THE OLDEST-OLD

As for AD in young elderly, the etiology of dementia in the oldest-old is unknown. Several genetic and environmental factors have been proposed to increase the risk of dementia in the oldest-old. Importantly, risk/protective factors for dementia in younger elderly subjects may not pertain to the oldest-old. Moreover, postmortem studies suggest that neuropathology is abundant in the oldest-old brains, and not necessarily correlated with dementia, making the determination of the etiology a difficult mission. In this section we review the risk factors and neurobiology of dementia in the oldest-old.

### Risk Factors for Dementia in the Oldest-Old

#### Age

The high rates of incidence and prevalence of dementia in the oldest-old indicate that age is an important risk factor. Although it has been suggested that dementia is an inevitable part of aging,^30^ dementia could result from the continued accumulation of potentially preventable age-related risk factors,^21^ eventually surpassing a threshold after which protective mechanisms (such as neuroimmune response) and compensatory facilities (such as reserve capacity) cannot maintain healthy cognition. Since aging is inevitable, managing modifiable risk factors could, at least partially, prevent or delay some of the devastating aspects of extremely old-age dementia.

#### Estrogen and estrogen therapy

Women’s life expectancy is longer than men’s. Also, sex differences in incidence/prevalence of all-cause dementia, as well as AD and VaD, have been reported in the oldest-old. Results from the 90+ Study suggested higher prevalence (all cases) of all-cause dementia in women than in men,^31^ although the incidence (“new” cases) rates were similar in both sexes.^22^ The authors suggested that sex differences in prevalence are due to shorter survival of men after a diagnosis of dementia, as previously reported in younger elderly.^32^ Examining dementia subtypes, the majority of the reports are in agreement with higher prevalence and incidence rates of AD in extremely old women.^19^,^26^,^28^,^33^–^36^ As for VaD, however, higher prevalence and incidence rates in very old men were suggested in some studies,^26^,^27^ but not all.^25^,^35^,^36^ One immediate suspect among gender differences is estrogen, the primary female gonadal hormone. Unlike testosterone, the primary male gonadal hormone, which gradually and moderately decreases with male aging,^37^ estrogen production in women ceases suddenly around the time of menopause.^38^ Indeed, epidemiological evidence has linked the loss of estrogen with an increased risk for the development of AD, and suggested that estrogen replacement would significantly decrease the incidence of AD (for reviews see^39^,^40^). Despite the majority of epidemiological and basic research that suggest beneficial actions of estradiol, some clinical trials examining the role of hormone replacement therapy in the development of AD, including the Women’s Health Initiative (WHI), have provided conflicting results.^41^–^43^ Finally, it has been suggested that women are at greater risk for dementia and AD simply because they live longer, and thus more likely to develop age-related disorders. A support for this notion came from the Leisure World Cohort Study, which suggested that estrogen therapy is associated with longevity, rather than dementia.^44^

#### Genes

To date, no clear evidence has shown an association between genetic factors and dementia in the oldest-old. This may seem at odds, since the genetic factors that are most consistently associated with dementia in younger elderly (particularly AD)^45^ and longevity^46^ are all related to a specific family of proteins—the lipoproteins. These genetic factors include: 1) the ε4 allele of apolipoprotein E (ApoE) gene that has been independently associated with increased risk of late-onset (age ≥ 65) AD^47^,^48^ and reduced chance of becoming a centenarian;^49^ and the genes for 2) microsomal transfer protein (mediates the rate-limiting step in lipoprotein synthesis); and 3) cholesteryl ester transfer protein (affects HDL and LDL particle size), which have been associated with longevity.^50^,^51^ None of these genes were associated with dementia in the oldest-old. In fact, the presence of the ApoE ε4 allele seems to lose its significance in predicting AD as age progresses.^52^ The lack of associations between dementia and lipoproteins in very old age add further evidence to the hypothesis that the oldest-old are likely to be biologically different from the younger-old.

#### Physical activity

In studies of younger elderly, physical activity has consistently been associated with decreased risk of dementia.^53^–^56^ A possible explanation is that physically active individuals are more resistant to adverse risk factor changes, which modulate the risk of dementia, such as diabetes or diabetes-like metabolic disorders (reviewed in^57^,^58^) and cardiovascular diseases (reviewed in 59,60). Other mechanisms may involve direct influences of physical activity on brain plasticity^61^ and structural and functional brain reserves.^62^ In addition, experimental studies have shown that exercise resulted in a decrease in Aβ plaques in the cerebral cortex and hippocampus in a mouse model for AD,^63^ and promoted hippocampal neurogenesis in a mouse model for amyotrophic lateral sclerosis.^64^ Although similar associations between physical activity and dementia may be expected in the oldest-old, such evidence is extremely scarce. Preliminary analyses of the 90+ Study showed that impairment in measures of physical performance (such as timed walking, balance, and hand grip) were associated with increased risk of dementia.^6^ Nevertheless, data of the 90+ Study from the 1980s associated late-life exercise with longevity, but not dementia.^65^ In order to assess fully the contribution of physical activity to risk of dementia in the oldest-old, exercise and activeness should be objectively evaluated in real time, years before the onset of dementia. This requires long prospective studies, which are currently unavailable.

#### Lifestyle

Similar to physical activity, other lifestyle-related factors have been associated with longevity. Those factors include eating habits reflected in body mass index (both being underweight and being obese increased the risk of mortality),^66^ alcohol consumption (more than 2 drinks per day reduced the risk of death by 15%),^67^ and caffeine intake (with a U-shaped mortality curve).^68^ None of these factors, however, were associated with prevalent dementia in the oldest-old.^6^

In summary, many of the risk and protective factors for dementia in the young elderly are not relevant for the oldest-old. Out of the reviewed factors, only age was consistently associated with dementia in the oldest-old. Estrogen showed some association with dementia in the oldest, but this association was not consistent through all studies and dementia subtypes. The other factors—the ε4 allele of the ApoE gene, physical activity, and healthy lifestyle—which were all associated with dementia in younger elderly, were not associated with dementia in the oldest-old. This difference supports the potential for differential neurobiology of AD and dementia in the oldest-old.

### Neurobiological Changes in Dementia of the Oldest-Old

“Dementia” is a general term for a group of disorders, and the distinction between dementia subtypes is largely dependent on their underlying neuropathology. Hence, for the most part, the following discussion describes the associations between pathologies of specific dementia subtypes and the clinical manifestation of general dementia symptoms. The major pathological hallmarks of AD, extracellular deposits of amyloid protein which form neuritic plaques and intraneuronal neurofibrillary tangles, are found with increasing frequency in advancing age.^69^ The age-related increases in AD pathologies, together with the increased incidence rates of dementia with age, suggest that the two are related. Recent studies, however, have shown that the association between the pathological features of AD and dementia is stronger in younger persons than in the oldest-old.^4^ This lack of association was found to be due to both high prevalence of cerebral pathologies in some non-demented oldest-old^70^–^74^ and low prevalence of these pathologies in many demented oldest-old.^75^–^78^ Moreover, in the oldest-old, an ApoE ε2 allele—which is considered protective against AD—was associated with a somewhat reduced risk of dementia, despite its association with increased AD neuropathology.^79^ Some of the above-mentioned studies (e.g.^4^,^74^,^75^) found these weaker relationships in the oldest-old not only for AD pathology, but also for other types of neuropathologies (hippocampal sclerosis, atrophy, vascular dementia, and diffuse Lewy body disease). Consistent with that, cerebrovascular pathologies, such as small-vessel disease and/or infarcts, were strongly associated with dementia in younger elderly but not in the oldest-old.^4^

Contrary to these findings, a recent study from the Baltimore Longitudinal Study of Aging found that plaques and tangles were significant predictors of dementia independent of age.^80^ This study also found that in participants older than 90 years of age, intracranial atherosclerosis predicted dementia in subjects with low Alzheimer’s pathology scores. A study of a relatively large number of autopsies found that mixed AD pathology and vascular pathology accounted for most dementia cases in very old persons.^81^ The cumulative effects of AD-type pathologies and vascular pathologies on cognition have been demonstrated in several studies.^82^,^83^

Another feature of aging and dementia is synaptic protein loss, which may dissociate oldest-old individuals with and without dementia. Head and colleagues studied several synaptic proteins in the frontal cortex of aged individuals (92–105 years) with a range of cognitive function. Synaptophysin protein levels were lower in individuals with dementia and correlated with cognitive function scores.^84^ The investigators concluded that these protein levels may protect neuronal function in oldest-old individuals and reflect compensatory responses that may be involved with maintaining cognition. Similarly to these findings, we have also found that gene and protein expression levels of synaptic markers decrease in persons with dementia, regardless of age.^85^

This considerable discrepancy between pathology and dementia in the oldest-old has focused attention on the importance of neuronal loss, rather than the accumulation of abnormal protein deposits, in causing cognitive impairment. Contrary to the traditional view, it now appears that neuron loss is restricted in normal brain aging and unlikely to account for age-related impairment of neocortical and hippocampal functions.^86^ Consistent with this idea, Savva et al. found that neocortical cerebral atrophy maintained a relationship with dementia across all age groups.^4^ The value of cerebral atrophy in predicting dementia was supported by *in vivo*^87^,^88^ and postmortem^89^ magnetic resonance imaging (MRI) studies. Specifically, a study from our group showed reduced functional MRI (fMRI) activation in highly functioning nonagenarians during a recognition memory task, as compared to younger subjects, suggesting effective usage of cognitive reserve.^90^ The association between neuronal loss and cognitive impairment, and the lack of association between AD/vascular pathologies and cognitive impairment, has led to the construct of “cognitive reserve,” the hypothesized capacity of the mature adult brain to resist the effects of disease or injury that would otherwise cause dementia.^91^ According to this hypothesis, elderly individuals with a high level of cognitive reserve may remain dementia-free in spite of the neuropathological changes. Several factors that predict lower risk of dementia, including high-quality education, occupational complexity, and balanced diet, were also associated with the biological advantage of cognitive ability, i.e. cognitive reserve (reviewed in^91^). A recent study by Murray et al. demonstrates that the magnitude of the contribution of education to cognitive function is greater than the negative impact of either of the two neuropathological burdens alone,^92^ emphasizing the role of both neuronal loss and neuronal reserve in the dementing processes of the oldest-old.

## SYMPTOMATOLOGY OF DEMENTIA IN THE OLDEST-OLD

### Cognitive Decline

Even without a “proper” dementia diagnosis, it is generally accepted that—on average—a gradual age-related cognitive decline occurs in humans, as well as non-human primates.^93^,^94^ Cognitive performance is a term that describes the composite outcome of multiple cognitive domains and the interactions between them. Therefore, “cognitive decline” may be the result of impairment in an individual domain or impairment in multiple domains, possibly to different extents. Studies have described age-related declines in many of the cognitive domains, including divided attention,^95^ verbal memory,^96^ working memory,^97^ and learning.^98^,^99^ Nevertheless, it appears that during normal aging, some domains are more susceptible to impairment than others. In particular, executive function and mental speed have been suggested as such vulnerable domains.^100^,^101^

This poses a new challenge on determining diagnosis of AD and other forms of dementia in the oldest-old. In spite of the great development in neuroimaging techniques such as MRI and positron emission tomography (PET), neuropsychological assessment remains the key instrument in diagnosing dementia and monitoring cognitive decline.

Several valid and reliable neuropsychological dementia screening instruments have been developed to address the issue of clinical dementia diagnosis in elderly in whom cognitive decline is expected. Those instruments include the Blessed Dementia Scale,^102^ Dementia Rating Scale,^103^ Mini-Mental State Examination (MMSE),^104^ and Modified Mini-Mental State Examination (3MS)—an expanded version of the MMSE.^105^ The MMSE is probably the most widely used, easy to administrate, cognitively comprehensive, and validated instrument for detecting dementia.^106^,^107^ It has also been validated in the oldest-old, as it was found to have good sensitivity and specificity across all age and educational groups.^108^

According to the National Institute of Neurological and Communicative Disorders and Stroke–Alzheimer’s Disease and Related Disorders Association (NINCDS-ADRDA), in order to fulfill research criteria for probable AD, a patient must 1) meet the core clinical criterion A—significant episodic memory impairment; 2) meet at least one of the supportive biomarker criteria—medial temporal lobe atrophy (criterion B), abnormal cerebrospinal fluid biomarker (criterion C), specific pattern on functional neuroimaging with PET (criterion D), or proven AD autosomal dominant mutation within the immediate family (criterion E); and 3) all other possible medical, psychiatric, and neurological explanations for the symptoms have been ruled out.^109^ Strikingly, these criteria are pertinent only to individuals below the age of 90. Given the age-related cognitive decline described above, it is essential to set norms suitable for the oldest-old in order to make a reliable diagnosis. Using the 90+ study, Whittle et al. compiled a relatively brief test battery for multiple cognitive domains, with an average time to complete of one hour.^110^ This study found that in non-demented oldest-old, cognitive performance declined with age for two-thirds of the tests, and a high prevalence (34%) of cognitive impairment was observed in a sample of non-demented oldest-old in another study from the same group.^111^ Studies from our group demonstrated that declines in cognitive performance are found even when comparing individuals aged 85–89 to those aged 90+ years,^112^ and that the rate of cognitive decline is faster in questionably demented nonagenarians compared to younger groups.^113^

Similarly, cross-sectional studies have compared cognitive abilities of disease-free 100-year-olds to those of younger age groups. For instance, Poon et al. found that centenarians performed significantly lower on verbal and performance measures than 60- to 80-year-olds but did not differ in their ability to solve practical problems.^114^ Similar findings were reported with Swedish centenarians on new learning and working memory tests compared with 16- to 57-year-olds.^115^

In conclusion, the oldest-old have lower cognitive functioning and faster cognitive decline than younger elderly, and this decline affects cognition globally.

### Sensory and Motor Disability

It seems to be commonly understood that very old people suffer from sensory losses and reduced physical and motor abilities. Extremely old individuals are typically portrayed in movies as having hunched walk, thick glasses, and loud speech, and replying with “What?!?” shouts to every question. This stereotypical presentation of old age is not detached from reality. Visual losses are frequent in the oldest-old, with prevalence of 59%.^116^ One of the most prevalent and debilitating types of vision loss is age-related macular degeneration, with 16.4% of white American, Australian, and European women aged 80 and older having the condition.^117^ Even more striking is the prevalence of hearing loss in this population. A large population-based study found that 90% of people aged 80–92 had some level of hearing loss.^118^ Results from the 90+ Study showed that 72% of the participants had significant hearing loss, vision loss, or both.^119^

As cognitive decline and dementia are very common in the oldest-old (as described above), it has been suggested that sensory disabilities play a role in cognitive functions of the very old. For instance, greater hearing loss was associated with the severity of cognitive dysfunction in a dose-response manner, in both demented patients and normal controls.^120^ However, hearing and vision losses may “mechanically” interfere in cognitive performances during neuropsychological evaluations, and result in false-positive classification of impaired performances as dementia. Gussekloo et al. reported that although both hearing impairment (prevalence 85%) and visual impairment (prevalence 59%) were associated with global cognitive impairment, only visual impairments were also associated with poorer scores on memory and cognitive speed, as measured with visually presented cognitive tests.^116^ This suggests practical disadvantage of sensory impairment during cognitive assessments. In order to compensate for vision and hearing loss when assessing the oldest-old, using their experience in the 90+ Study, Brumback-Peltz et al. suggested some standardized changes in administration methods.^29^ These changes include providing amplified auditory and visual stimuli, and modifying common neuropsychological tests to include large type-face cards that are presented simultaneously with auditory tasks, spoken in a loud, clear voice.^29^

On the other hand, some studies suggest that sensory loss, especially vision impairment, is truly biologically associated with cognitive decline. It has been shown that poor vision precedes dementia,^121^ and vision impairment predicts cognitive decline,^122^ even when evaluated using the blind version of MMSE.^123^ An underlying biological mechanism for this association is suggested by several lines of evidence: AD patients often have retinal nerve degenerative changes,^124^ caused by reduced numbers of ganglion cells and axons^125^,^126^ and retinal amyloid plaques accumulation.^127^ In addition, diabetic retinopathy has been associated with cognitive decline.^128^ Several studies further suggested that treatment of specific visual disorders could alleviate cognitive decline,^121^,^129^,^130^ although caution must be applied when interpreting the treatment effect as “causative.” Nevertheless, postoperative increase in visual cortex grey matter volume was observed after cataract surgery,^131^ and disrupting retinoid signaling pathway in rats resulted in loss of choline acetyl transferase expression and amyloid β deposition in cerebral blood vessels,^132^ suggesting that the relationship between visual impairment and cognitive decline is not merely a mechanical artifact.

Similarly to cognition and sensory abilities, motor abilities (as speed and power) and physical performance also decline with age. Moreover, it appears that these motor and physical declines may be associated with declines in cognition^133^ and an increased risk of dementia, disability, and death. Earlier we have reviewed studies that suggest physical activity may be protective against dementia and cognitive decline. However, the physical and motor disabilities that are common in the oldest-old population are likely to prevent them from performing physical activity. These disabilities may well precede cognitive decline, and therefore may reflect common pathways in age-associated mechanisms of physical and cognitive decline.

### Disability and Activities of Daily Living

Comorbidity is very common among the oldest-old. Suffering from neurodegenerative disorder or related medical illness may result in difficulties in carrying out every-day activities. The 90+ Study found that almost all participants had at least one major medical illness or cardiovascular risk factor, and 62% had two or more.^134^ In centenarians it was found that, on average, they had more than four chronic conditions or diseases.^135^

Physical disability, medical illness, and cognitive impairment can all contribute to functional disability, presented as functional losses in activities such as driving and managing financial matters. Therefore, functional disability is expected to be very prevalent in the oldest-old. A study of people aged 84–90 found a minority (23%) of high-functioning subjects with no or only mild disability.^136^ In addition, the 90+ Study found that, overall, 16.4% became disabled each year, and that the disability incidence increased with age from 8.3% in the 90–94 age group to 25.7% in the 95 years and older age group.^134^

A widely accepted measure of disability is the index of Activities of Daily Living (ADL), including basic ADLs (BADLs)^137^ and instrumental ADLs (IADLs).^138^ Grades of the BADLs summarize overall performance in self-care tasks, such as bathing, dressing, using the toilet, transferring, continence, and feeding. IADLs include tasks such as housework, taking medication as prescribed, managing money, shopping for groceries, and using technology. IADLs consist of tasks which are not necessary for fundamental functioning, but they let an individual live independently in a community.^139^ IADL independence is one of the defining features that distinguishes normal aging from mild cognitive impairment (MCI) and dementia,^140^ whereas losses in the ability to perform BADL are characteristics of moderate to severe dementia.^141^ For instance, a positive relationship has been observed between the level of cognitive impairment and the decline in IADLs such as managing money, telephone use, preparing meals, and medications.^142^ A recent study found that lower Dementia Rating Scale scores were associated with greater reported difficulties and impairments in ADLs, as follows: 1) participants in the “mild” range of cognitive impairment were most likely to have difficulties with IADLs such as household upkeep, managing finances, and functioning outside a familiar environment; 2) a large proportion of individuals with “moderate” cognitive impairment reported difficulty with washing/grooming and dressing (BADLs), and additionally reported difficulty to some degree in all of the IADLs; 3) individuals in the “severe” cognitive impairment range were likely to have difficulties with all BADLs, and over 85% of the severe group reported difficulty in all IADLs.^143^

Losses in the ability to perform ADLs are very common in the oldest-old. Difficulty in one or more BADLs was present in 71% of 90–94-year-olds, 89% of 95–99-year-olds, and 97% of centenarians, with walking as the BADL most commonly causing difficulty (70%), and bathing as the BADL most commonly causing dependency (51%).^144^ Bathing is described as a “sentinel event in the disabling process,”^145^ and those unable to bathe themselves without help are more likely to need long-term care.^146^ In what seems to be a conflicting result, a recent publication of the Newcastle 85+ study reported that, of the different ADLs, “cutting toenails” was the first item with which participants had difficulty and “feeding” the last.^147^ In this study, however, the results rely on self-reports, indicating that the study population consisted of higher-performing individuals. There is scarce information on the extent of the contribution of ADL and IADL to oldest-old dementia.

Functional disabilities which extend beyond the specified ADLs have also been associated with aging and dementia. Fine hand motor function (e.g. precision pinch) and gross hand motor function (e.g. pinch and grip force) decline with age^148^ and are associated with MCI and, to a larger extent, with AD (reviewed in^149^). Impairment in hand-motor activity is likely to contribute to the high prevalence of difficulties in performing IADLs observed in the oldest-old.

## DISCUSSION

The increase in the proportion of the oldest-old in the Western population and the increased prevalence of dementia in this age group emphasize the importance of giving extra attention to investigating its specific characteristics. This is not an easy task, since the majority of the oldest-old suffer from many medical conditions, age-related cognitive decline, sensory and motor disabilities, and disabilities in performing everyday activities. This group also presents neurobiological features which differ from younger elderly, including great variability, making interpretation of their contribution to dementia more complex. To complicate characterization further, risk factors for dementia in the oldest-old do not seem to comply with those in young elderly, with age being the only significant risk factor.

These differences raise the question whether the oldest-old are a select population, predisposed to longevity by a veiled biological mechanism, and promoted by modern healthcare. If so, normal aging processes cannot be inferred by investigating the oldest-old, and dementing processes in the oldest-old cannot be inferred from those in young elderly. Consistent with the notion of selected population, the “compression of morbidity” hypothesis proposes that individuals who reach the limits of the human life-span compress the onset and duration of illnesses toward the end of life.^150^ It has been shown that over 83% of centenarians delayed (to their ninth decade or later) or escaped the most lethal diseases of the elderly population, i.e. heart disease, non-skin cancer, and stroke.^151^ Moreover, “delayers” and “escapers” may be two distinct populations. Escaping lethal diseases by the age of 100 suggests an innate advantage, a “fountain of youth” sort of mechanism, which acts throughout life from early development. Richard Cutler, in his classic paper in gerontology, proposed that persons who achieve extreme old age have genetic variations that affect the basic mechanisms of aging and promote a decreased susceptibility to age-associated diseases.^152^ The decreased susceptibility may be due to the absence of “disease genes,”^153^ or due to the presence of “longevity-enabling genes” that confer protection against the basic mechanisms of aging or age-related illnesses.^46^ In support of this notion, evidence from studies of centenarian pedigrees showed that their family members are more likely to have such combinations of factors in common than the general population, as they had much lower death rates than those of the general population (reviewed in^154^). The genetic and neurobiological composition of the “escapers” is therefore unique and may present a basis for investigations of protective factors for healthy aging and cognition. Since, overall, the data on the oldest old, and particularly on dementia, are scarce, interpretations must be made with caution.

Achieving exceptional longevity by delaying age-related diseases, however, offers a much less dramatic approach. In this approach, different levels of risk factors, some of them potentially modifiable, will determine the individual’s probability of remaining in good health when others of this age group succumb to illness.

By itself, the notion of delaying or escaping diseases until exceptional old age cannot explain the difficulty in characterizing the etiology of dementia in the oldest-old. The principle of demographic selection dictates that the oldest-old are more similar to one another, genetically and environmentally, than younger elderly individuals, where, theoretically, more heterogeneity is evident. This appears to contradict the great variability in neurobiological features observed in this age group. However, in the oldest-old, the biological phenotypes are only weakly associated with cognition,^4^ possibly reflecting age-related accumulation of varied biological features. As the oldest-old group is probably composed of individuals who are genetically resilient (“escapers”) or with low levels of risk factors (“delayers”) for developing disease, it is with reason that dementing processes will be manifested at levels of pathologies which are different than those of younger elderly. In delayers, low levels of risk factors will result in a low rate of accumulation of pathologies, eventually surpassing the threshold for cognitive decline. In these individuals, the association between cognitive decline and neuropathological features is expected to resemble the association in younger elderly. In escapers on the other hand, the composition, rather than amount, of the accumulated pathologies is likely to play a bigger role in cognition—a large variety of minimal pathologies (each one by itself is not sufficient for causing dementia) will trigger the dementing processes. The greater proportion of resilient individuals in the oldest-old, compared to younger population, may account for the diminished association between pathology and cognition. The fact that not all oldest-old are necessarily resilient, may explain the discrepancies in findings in different studies.

To date, research of the oldest-old is limited not only by the medical and physical features of extreme age, but also by administrative considerations. The NINCDS gold standard for AD clinical diagnostic criteria are limited to age 90,^155^ leading to exclusion of those at highest risk from major international studies. Thus, raising the awareness of the clinical and pathological meaning of dementia in the oldest-old is of enormous urgency. Only extensive research will enable us to provide this rapidly growing population with good quality of life and graceful aging.

## Abbreviations:

AD: Alzheimer’s disease;
ADL: Activities of Daily Living;
ApoE: apolipoprotein E;
BADL: basic ADL;
IADL: instrumental ADL;
MCI: mild cognitive impairment;
MMSE: Mini-Mental State Examination;
MRI: magnetic resonance imaging;
NINCDS: National Institute of Neurological and Communicative Disorders and Stroke—Alzheimer’s Disease;
PET: positron emission tomography;
VaD: vascular dementia.

## References

[1] World Health Organization http://tinyurl.com/cjx9eqa.

[2] United States Census Bureau http://tinyurl.com/4sv5j2.

[3] United Nations, Department of Economic and Social Affairs, Population
Division http://tinyurl.com/bngtvgz.

[4] Savva GM, Wharton SB, Ince PG, Forster G, Matthews FE, Brayne C (2009). Age, neuropathology, and dementia. N Engl J Med.

[5] Poon LW, Woodard JL, Stephen Miller L (2012). Understanding dementia prevalence among centenarians. J Gerontol A BiolSci Med Sci.

[6] Kawas CH (2008). The oldest old and the 90+ Study. Alzheimers Dement.

[7] Werner CA http://2010.census.gov/2010census/.

[8] (2000). American Psychiatric Association Diagnostic and Statistical Manual of Mental
Disorders, Fourth Edition, Text Revision (DSM-IV-TR).

[9] Thomassen R, van Schaick HW, Blansjaar BA (1998). Prevalence of dementia over age 100. Neurology.

[10] Andersen-Ranberg K, Vasegaard L, Jeune B (2001). Dementia is not inevitable: a population-based study of Danish
centenarians. J Gerontol B Psychol Sci Soc Sci.

[11] Silver MH, Jilinskaia E, Perls TT (2001). Cognitive functional status of age-confirmed centenarians in a
population-based study. J Gerontol B Psychol Sci Soc Sci.

[12] Hagberg B, Bauer Alfredson B, Poon LW, Homma A (2001). Cognitive functioning in centenarians: a coordinated analysis of
results from three countries. J Gerontol B Psychol Sci Soc Sci.

[13] Silver M, Newell K, Hyman B, Growdon J, Hedley-Whyte ET, Perls T (1998). Unraveling the mystery of cognitive changes in old age: correlation of
neuropsychological evaluation with neuropathological findings in the extreme
old. Int Psychogeriatr.

[14] Green MS, Kaye JA, Ball MJ (2000). The Oregon brain aging study: neuropathology accompanying healthy
aging in the oldest old. Neurology.

[15] Hagnell O, Ojesjo L, Rorsman B (1992). Incidence of dementia in the Lundby Study. Neuroepidemiology.

[16] Fichter MM, Schroppel H, Meller I (1996). Incidence of dementia in a Munich community sample of the oldest
old. Eur Arch Psychiatry Clin Neurosci.

[17] The Canadian Study of Health and Aging Working Group (2000). The incidence of dementia in Canada. Neurology.

[18] Ruitenberg A, Ott A, van Swieten JC, Hofman A, Breteler MM (2001). Incidence of dementia: does gender make a difference?. Neurobiol Aging.

[19] Edland SD, Rocca WA, Petersen RC, Cha RH, Kokmen E (2002). Dementia and Alzheimer disease incidence rates do not vary by sex in
Rochester, Minn. Arch Neurol.

[20] Miech RA, Breitner JC, Zandi PP, Khachaturian AS, Anthony JC, Mayer L (2002). Incidence of AD may decline in the early 90s for men, later for women:
the Cache County study. Neurology.

[21] Hall CB, Verghese J, Sliwinski M (2005). Dementia incidence may increase more slowly after age 90: results from
the Bronx Aging Study. Neurology.

[22] Corrada MM, Brookmeyer R, Paganini-Hill A, Berlau D, Kawas CH (2010). Dementia incidence continues to increase with age in the oldest old:
the 90+ Study. Ann Neurol.

[23] Jorm AF, Jolley D (1998). The incidence of dementia: a meta-analysis. Neurology.

[24] Peltz CB, Corrada MM, Berlau DJ, Kawas CH (2011). Incidence of dementia in oldest-old with amnestic MCI and other
cognitive impairments. Neurology.

[25] Knopman DS, Rocca WA, Cha RH, Edland SD, Kokmen E (2002). Incidence of vascular dementia in Rochester, Minn,
1985–1989. Arch Neurol.

[26] Lopez-Pousa S, Vilalta-Franch J, Llinas-Regla J, Garre-Olmo J, Roman GC (2004). Incidence of dementia in a rural community in Spain: the Girona cohort
study. Neuroepidemiology.

[27] Kukull WA, Higdon R, Bowen JD (2002). Dementia and Alzheimer disease incidence: a prospective cohort
study. Arch Neurol.

[28] Fratiglioni L, Viitanen M, von Strauss E, Tontodonati V, Herlitz A, Winblad B (1997). Very old women at highest risk of dementia and Alzheimer’s
disease: incidence data from the Kungsholmen Project, Stockholm. Neurology.

[29] Brumback-Peltz C, Balasubramanian AB, Corrada MM, Kawas CH (2011). Diagnosing dementia in the oldest-old. Maturitas.

[30] Drachman DA (1994). If we live long enough, will we all be demented?. Neurology.

[31] Corrada MM, Brookmeyer R, Berlau D, Paganini-Hill A, Kawas CH (2008). Prevalence of dementia after age 90: results from the 90+
Study. Neurology.

[32] Waring SC, Doody RS, Pavlik VN, Massman PJ, Chan W (2005). Survival among patients with dementia from a large multi-ethnic
population. Alzheimer Dis Assoc Disord.

[33] Letenneur L, Gilleron V, Commenges D, Helmer C, Orgogozo JM, Dartigues JF (1999). Are sex and educational level independent predictors of dementia and
Alzheimer’s disease? Incidence data from the PAQUID
project. J Neurol Neurosurg Psychiatry.

[34] Andersen K, Launer LJ, Dewey ME (1999). Gender differences in the incidence of AD and vascular dementia: the
EURODEM Studies. EURODEM Incidence Research Group. Neurology.

[35] Fratiglioni L, Launer LJ, Andersen K (2000). Incidence of dementia and major subtypes in Europe: A collaborative
study of population-based cohorts. Neurologic Diseases in the Elderly Research
Group. Neurology.

[36] Lobo A, Launer LJ, Fratiglioni L (2000). Prevalence of dementia and major subtypes in Europe: a collaborative
study of population-based cohorts. Neurologic Diseases in the Elderly Research
Group. Neurology.

[37] Harman SM, Metter EJ, Tobin JD, Pearson J, Blackman MR (2001). Longitudinal effects of aging on serum total and free testosterone
levels in healthy men. Baltimore Longitudinal Study of Aging. J Clin Endocrinol Metab.

[38] Burger HG, Dudley EC, Robertson DM, Dennerstein L (2002). Hormonal changes in the menopause transition. Recent Prog Horm Res.

[39] Cholerton B, Gleason CE, Baker LD, Asthana S (2002). Estrogen and Alzheimer’s disease: the story so
far. Drugs Aging.

[40] Henderson VW (1997). The epidemiology of estrogen replacement therapy and
Alzheimer’s disease. Neurology.

[41] Brenner DE, Kukull WA, Stergachis A (1994). Postmenopausal estrogen replacement therapy and the risk of
Alzheimer’s disease: a population-based case-control study. Am J Epidemiol.

[42] Shumaker SA, Legault C, Kuller L (2004). Conjugated equine estrogens and incidence of probable dementia and
mild cognitive impairment in postmenopausal women: Women’s Health
Initiative Memory Study. JAMA.

[43] Shumaker SA, Legault C, Rapp SR (2003). Estrogen plus progestin and the incidence of dementia and mild
cognitive impairment in postmenopausal women: the Women’s Health
Initiative Memory Study: a randomized controlled trial. JAMA.

[44] Paganini-Hill A, Corrada MM, Kawas CH (2006). Increased longevity in older users of postmenopausal estrogen therapy:
the Leisure World Cohort Study. Menopause.

[45] Cacabelos R, Martinez-Bouza R (2011). Genomics and pharmacogenomics of dementia. CNS Neurosci Ther.

[46] Perls T, Kunkel LM, Puca AA (2002). The genetics of exceptional human longevity. J Am Geriatr Soc.

[47] Corder EH, Saunders AM, Strittmatter WJ (1993). Gene dose of apolipoprotein E type 4 allele and the risk of
Alzheimer’s disease in late onset families. Science.

[48] Evans DA, Beckett LA, Field TS (1997). Apolipoprotein E epsilon4 and incidence of Alzheimer disease in a
community population of older persons. JAMA.

[49] Schachter F, Faure-Delanef L, Guenot F (1994). Genetic associations with human longevity at the APOE and ACE
loci. Nat Genet.

[50] Geesaman BJ, Benson E, Brewster SJ (2003). Haplotype-based identification of a microsomal transfer protein marker
associated with the human lifespan. Proc Natl Acad Sci U S A.

[51] Barzilai N, Atzmon G, Schechter C (2003). Unique lipoprotein phenotype and genotype associated with exceptional
longevity. JAMA.

[52] Breitner JC, Wyse BW, Anthony JC (1999). APOE-epsilon4 count predicts age when prevalence of AD increases, then
declines: the Cache County Study. Neurology.

[53] Wang L, Larson EB, Bowen JD, van Belle G (2006). Performance-based physical function and future dementia in older
people. Arch Intern Med.

[54] Laurin D, Verreault R, Lindsay J, MacPherson K, Rockwood K (2001). Physical activity and risk of cognitive impairment and dementia in
elderly persons. Arch Neurol.

[55] Buchman AS, Boyle PA, Yu L, Shah RC, Wilson RS, Bennett DA (2012). Total daily physical activity and the risk of AD and cognitive decline
in older adults. Neurology.

[56] Abbott RD, White LR, Ross GW, Masaki KH, Curb JD, Petrovitch H (2004). Walking and dementia in physically capable elderly men. JAMA.

[57] de la Monte SM (2012). Brain insulin resistance and deficiency as therapeutic targets in
Alzheimer’s disease. Curr Alzheimer Res.

[58] Ravona-Springer R, Schnaider-Beeri M (2011). The association of diabetes and dementia and possible implications for
nondiabetic populations. Expert Rev Neurother.

[59] Kovacic JC, Castellano JM, Fuster V (2012). The links between complex coronary disease, cerebrovascular disease,
and degenerative brain disease. Ann N Y Acad Sci.

[60] Gorelick PB, Scuteri A, Black SE (2011). Vascular contributions to cognitive impairment and dementia: a
statement for healthcare professionals from the American Heart
Association/American Stroke Association. Stroke.

[61] Cotman CW, Berchtold NC (2002). Exercise: a behavioral intervention to enhance brain health and
plasticity. Trends Neurosci.

[62] Scarmeas N, Zarahn E, Anderson KE (2003). Association of life activities with cerebral blood flow in Alzheimer
disease: implications for the cognitive reserve hypothesis. Arch Neurol.

[63] Adlard PA, Perreau VM, Pop V, Cotman CW (2005). Voluntary exercise decreases amyloid load in a transgenic model of
Alzheimer’s disease. J Neurosci.

[64] Ma X, Hamadeh MJ, Christie BR, Foster JA, Tarnopolsky MA (2012). Impact of treadmill running and sex on hippocampal neurogenesis in the
mouse model of amyotrophic lateral sclerosis. PLoS One.

[65] Kawas C, Corrada M, Paganini-Hill A (2003). Low weight in early adulthood and later life exercise increase chances
of surviving to age 90. Neurology.

[66] Corrada MM, Kawas CH, Mozaffar F, Paganini-Hill A (2006). Association of body mass index and weight change with all-cause
mortality in the elderly. Am J Epidemiol.

[67] Paganini-Hill A, Kawas CH, Corrada MM (2007). Type of alcohol consumed, changes in intake over time and mortality:
the Leisure World Cohort Study. Age Ageing.

[68] Paganini-Hill A, Kawas CH, Corrada MM (2007). Non-alcoholic beverage and caffeine consumption and mortality: the
Leisure World Cohort Study. Prev Med.

[69] Braak H, Braak E (1997). Frequency of stages of Alzheimer-related lesions in different age
categories. Neurobiol Aging.

[70] Imho A, Kovari E, von Gunten A (2007). Morphological substrates of cognitive decline in nonagenarians and
centenarians: a new paradigm?. J Neurol Sci.

[71] Crystal H, Dickson D, Fuld P (1988). Clinico-pathologic studies in dementia: nondemented subjects with
pathologically confirmed Alzheimer’s disease. Neurology.

[72] Katzman R, Terry R, DeTeresa R (1988). Clinical, pathological, and neurochemical changes in dementia: a
subgroup with preserved mental status and numerous neocortical
plaques. Ann Neurol.

[73] Price JL, McKeel DW, Buckles VD (2009). Neuropathology of nondemented aging: presumptive evidence for
preclinical Alzheimer disease. Neurobiol Aging.

[74] Silve MH, Newell K, Brady C, Hedley-White ET, Perls TT (2002). Distinguishing between neurodegenerative disease and disease-free
aging: correlating neuropsychological evaluations and neuropathological studies in
centenarians. Psychosom Med.

[75] Crystal HA, Dickson D, Davies P, Masur D, Grober E, Lipton RB (2000). The relative frequency of “dementia of unknown
etiology” increases with age and is nearly 50% in
nonagenarians. Arch Neurol.

[76] Polvikoski T, Sulkava R, Myllykangas L (2001). Prevalence of Alzheimer’s disease in very elderly people: a
prospective neuropathological study. Neurology.

[77] Corrada M, Head E, Kim R, Kawas C (2005). Braak and Braak staging and dementia in the oldest-old: Preliminary
results from the 90+ Study. Neurology.

[78] Haroutunian V, Schnaider-Beeri M, Schmeidler J (2008). Role of the neuropathology of Alzheimer disease in dementia in the
oldest-old. Arch Neurol.

[79] Berlau DJ, Corrada MM, Head E, Kawas CH (2009). APOE epsilon2 is associated with intact cognition but increased
Alzheimer pathology in the oldest old. Neurology.

[80] Dolan D, Troncoso J, Resnick SM, Crain BJ, Zonderman AB, O’Brien RJ (2010). Age, Alzheimer’s disease and dementia in the Baltimore
Longitudinal Study of Ageing. Brain.

[81] Jellinger KA, Attems J (2010). Prevalence of dementia disorders in the oldest-old: an autopsy
study. Acta Neuropathol.

[82] Nagy Z, Esiri MM, Jobst KA (1997). The effects of additional pathology on the cognitive deficit in
Alzheimer disease. J Neuropathol Exp Neurol.

[83] Snowdon DA, Greiner LH, Mortimer JA, Riley KP, Greiner PA, Markesbery WR (1997). Brain infarction and the clinical expression of Alzheimer
disease. The Nun Study JAMA.

[84] Head E, Corrada MM, Kahle-Wrobleski K (2009). Synaptic proteins, neuropathology and cognitive status in the
oldest-old. Neurobiol Aging.

[85] Schnaider Beeri M, Haroutunian V, Schmeidler J (2012). Synaptic protein deficits are associated with dementia irrespective of
extreme old age. Neurobiol Aging.

[86] Morrison JH, Hof PR (1997). Life and death of neurons in the aging brain. Science.

[87] Henneman WJ, Sluimer JD, Barnes J (2009). Hippocampal atrophy rates in Alzheimer disease: added value over whole
brain volume measures. Neurology.

[88] Morra JH, Tu Z, Apostolova LG (2009). Automated mapping of hippocampal atrophy in 1-year repeat MRI data
from 490 subjects with Alzheimer’s disease, mild cognitive impairment, and
elderly controls. Neuroimage.

[89] Barkhof F, Polvikoski TM, van Straaten EC (2007). The significance of medial temporal lobe atrophy: a postmortem MRI
study in the very old. Neurology.

[90] Beeri MS, Lee H, Cheng H, Wollman D, Silverman JM, Prohovnik I (2011). Memory activation in healthy nonagenarians. Neurobiol Aging.

[91] Whalley LJ, Deary IJ, Appleton CL, Starr JM (2004). Cognitive reserve and the neurobiology of cognitive
aging. Ageing Res Rev.

[92] Murray AD, Staff RT, McNeil CJ (2011). The balance between cognitive reserve and brain imaging biomarkers of
cerebrovascular and Alzheimer’s diseases. Brain.

[93] Picq JL, Aujard F, Volk A, Dhenain M (2012). Age-related cerebral atrophy in nonhuman primates predicts cognitive
impairments. Neurobiol Aging.

[94] Salthouse TA (2003). Memory aging from 18 to 80. Alzheimer Dis Assoc Disord.

[95] McDowd JM, Craik FI (1988). Effects of aging and task difficulty on divided attention
performance. J Exp Psychol Hum Percept Perform.

[96] Shaw RJ, Craik FI (1989). Age differences in predictions and performance on a cued recall
task. Psychol Aging.

[97] Morris RG, Craik FI, Gick ML (1990). Age differences in working memory tasks: the role of secondary memory
and the central executive system. Q J Exp Psychol A.

[98] Petersen RC, Smith G, Kokmen E, Ivnik RJ, Tangalos EG (1992). Memory function in normal aging. Neurology.

[99] Youngjohn JR, Crook TH (1993). Learning, forgetting, and retrieval of everyday material across the
adult life span. J Clin Exp Neuropsychol.

[100] Salthouse TA (1996). The processing-speed theory of adult age differences in
cognition. Psychol Rev.

[101] Salthouse TA, Atkinson TM, Berish DE (2003). Executive functioning as a potential mediator of age-related cognitive
decline in normal adults. J Exp Psychol Gen.

[102] Blessed G, Tomlinson BE, Roth M (1968). The association between quantitative measures of dementia and of
senile change in the cerebral grey matter of elderly subjects. Br J Psychiatry.

[103] Mattis S (1988). Dementia Rating Scale: Professional Manual.

[104] Folstein MF, Folstein SE, McHugh PR (1975). “Mini-mental state”. A practical method for grading
the cognitive state of patients for the clinician. J Psychiatr Res.

[105] Teng EL, Chui HC (1987). The Modified Mini-Mental State (3MS) examination. J Clin Psychiatry.

[106] Tombaugh TN, McIntyre NJ (1992). The mini-mental state examination: a comprehensive
review. J Am Geriatr Soc.

[107] Malloy PF, Cummings JL, Coffey CE (1997). Cognitive screening instruments in neuropsychiatry: a report of the
Committee on Research of the American Neuropsychiatric Association. J Neuropsychiatry Clin Neurosci.

[108] Kahle-Wrobleski K, Corrada MM, Li B, Kawas CH (2007). Sensitivity and specificity of the mini-mental state examination for
identifying dementia in the oldest-old: the 90+ Study. J Am Geriatr Soc.

[109] Dubois B, Feldman HH, Jacova C (2007). Research criteria for the diagnosis of Alzheimer’s disease:
revising the NINCDS-ADRDA criteria. Lancet Neurol.

[110] Whittle C, Corrada MM, Dick M (2007). Neuropsychological data in nondemented oldest old: the 90+
Study. J Clin Exp Neuropsychol.

[111] Peltz CB, Corrada MM, Berlau DJ, Kawas CH (2012). Cognitive impairment in nondemented oldest-old: prevalence and
relationship to cardiovascular risk factors. Alzheimers Dement.

[112] Beeri MS, Schmeidler J, Sano M (2006). Age, gender, and education norms on the CERAD neuropsychological
battery in the oldest old. Neurology.

[113] Ravona-Springer R, Luo X, Schmeidler J (2011). The association of age with rate of cognitive decline in elderly
individuals residing in supporting care facilities. Alzheimer Dis Assoc Disord.

[114] Poon LW, Martin P, Clayton GM, Messner S, Noble CA, Johnson MA (1992). The influences of cognitive resources on adaptation and old
age. Int J Aging Hum Dev.

[115] Samuelsson SM, Alfredson BB, Hagberg B (1997). The Swedish Centenarian Study: a multidisciplinary study of five
consecutive cohorts at the age of 100. Int J Aging Hum Dev.

[116] Gussekloo J, de Craen AJ, Oduber C, van Boxtel MP, Westendorp RG (2005). Sensory impairment and cognitive functioning in oldest-old subjects:
the Leiden 85+ Study. Am J Geriatr Psychiatry.

[117] Friedman DS, O’Colmain BJ, Munoz B (2004). Prevalence of age-related macular degeneration in the United
States. Arch Ophthalmol.

[118] Cruickshanks KJ, Wiley TL, Tweed TS (1998). Prevalence of hearing loss in older adults in Beaver Dam, Wisconsin.
The Epidemiology of Hearing Loss Study. Am J Epidemiol.

[119] Kahle-Wrobleski K, Corrada N, Kawas C, Miller BL, Boeve BF (2009). Dementia and Cognition in the Oldest-old. The Behavioral Neurology of Dementia.

[120] Uhlmann RF, Larson EB, Rees TS, Koepsell TD, Duckert LG (1989). Relationship of hearing impairment to dementia and cognitive
dysfunction in older adults. JAMA.

[121] Rogers MA, Langa KM (2010). Untreated poor vision: a contributing factor to late-life
dementia. Am J Epidemiol.

[122] Lin MY, Gutierrez PR, Stone KL (2004). Vision impairment and combined vision and hearing impairment predict
cognitive and functional decline in older women. J Am Geriatr Soc.

[123] Reyes-Ortiz CA, Kuo YF, DiNuzzo AR, Ray LA, Raji MA, Markides KS (2005). Near vision impairment predicts cognitive decline: data from the
Hispanic Established Populations for Epidemiologic Studies of the
Elderly. J Am Geriatr Soc.

[124] Lu Y, Li Z, Zhang X (2010). Retinal nerve fiber layer structure abnormalities in early
Alzheimer’s disease: evidence in optical coherence
tomography. Neurosci Lett.

[125] Iser PK, Altinas O, Tokay T, Yuksel N (2006). Relationship between cognitive impairment and retinal morphological
and visual functional abnormalities in Alzheimer disease. J Neuroophthalmol.

[126] Kesler A, Vakhapova V, Korczyn AD, Naftaliev E, Neudorfer M (2011). Retinal thickness in patients with mild cognitive impairment and
Alzheimer’s disease. Clin Neurol Neurosurg.

[127] Koronyo-Hamaoui M, Koronyo Y, Ljubimov AV (2011). Identification of amyloid plaques in retinas from Alzheimer’s
patients and noninvasive in vivo optical imaging of retinal plaques in a mouse
model. Neuroimage.

[128] Ding J, Strachan MW, Reynolds RM (2010). Diabetic retinopathy and cognitive decline in older people with type 2
diabetes: the Edinburgh Type 2 Diabetes Study. Diabetes.

[129] Fagerstrom R (1992). Correlations of memory and learning with vision in aged patients
before and after a cataract operation. Psychol Rep.

[130] Tamura H, Tsukamoto H, Mukai S (2004). Improvement in cognitive impairment after cataract surgery in elderly
patients. J Cataract Refract Surg.

[131] Lou AR, Madsen KH, Julian HO (2011). Postoperative increase in grey matter volume in visual cortex after
unilateral cataract surgery. Acta Ophthalmol.

[132] Corcoran JP, So PL, Maden M (2004). Disruption of the retinoid signalling pathway causes a deposition of
amyloid beta in the adult rat brain. Eur J Neurosci.

[133] Wilson RS, Bienias JL, Evans DA, Bennett DA (2004). The Religious Orders Study: overview and relation between change in
cognitive and motor speed. Aging Neuropsychol Cogn.

[134] Berlau DJ, Corrada MM, Peltz CB, Kawas CH (2012). Disability in the oldest-old: incidence and risk factors in the
90+ Study. Am J Geriatr Psychiatry.

[135] Andersen-Ranberg K, Schroll M, Jeune B (2001). Healthy centenarians do not exist, but autonomous centenarians do: a
population-based study of morbidity among Danish centenarians. J Am Geriatr Soc.

[136] Zarit SH, Johansson B, Berg S (1993). Functional impairment and co-disability in the oldest
old. J Aging Health.

[137] Katz S, Ford AB, Moskowitz RW, Jackson BA, Jaffe MW (1963). Studies of illness in the aged. The index of ADL: a standardized
measure of biological and psychosocial function. JAMA.

[138] Lawton MP, Brody EM (1969). Assessment of older people: self-maintaining and instrumental
activities of daily living. Gerontologist.

[139] Bookman A, Harrington M, Pass L, Reisner E (2007). Family Caregiver Handbook.

[140] Gold DA (2012). An examination of instrumental activities of daily living assessment
in older adults and mild cognitive impairment. J Clin Exp Neuropsychol.

[141] Bullock R, Hammond G (2003). Realistic expectations: the management of severe Alzheimer
disease. Alzheimer Dis Assoc Disord.

[142] McGuire LC, Ford ES, Ajani UA (2006). Cognitive functioning as a predictor of functional disability in later
life. Am J Geriatr Psychiatry.

[143] Fields JA, Machulda M, Aakre J (2010). Utility of the DRS for predicting problems in day-to-day
functioning. Clin Neuropsychol.

[144] Berlau DJ, Corrada MM, Kawas C (2009). The prevalence of disability in the oldest-old is high and continues
to increase with age: findings from The 90+ Study. Int J Geriatr Psychiatry.

[145] Gill TM, Guo Z, Allore HG (2006). The epidemiology of bathing disability in older
persons. J Am Geriatr Soc.

[146] Gill TM, Allore HG, Han L (2006). Bathing disability and the risk of long-term admission to a nursing
home. J Gerontol A Biol Sci Med Sci.

[147] Kingston A, Collerton J, Davies K, Bond J, Robinson L, Jagger C (2012). Losing the ability in activities of daily living in the oldest old: a
hierarchic disability scale from the Newcastle 85+ study. PLoS One.

[148] Ranganathan VK, Siemionow V, Sahgal V, Yue GH (2001). Effects of aging on hand function. J Am Geriatr Soc.

[149] Scherder E, Dekker W, Eggermont L (2008). Higher-level hand motor function in aging and (preclinical) dementia:
its relationship with (instrumental) activities of daily life—a
mini-review. Gerontology.

[150] Vita AJ, Terry RB, Hubert HB, Fries JF (1998). Aging, health risks, and cumulative disability. N Engl J Med.

[151] Evert J, Lawler E, Bogan H, Perls T (2003). Morbidity profiles of centenarians: survivors, delayers, and
escapers. J Gerontol A Biol Sci Med Sci.

[152] Cutler RG (1975). Evolution of human longevity and the genetic complexity governing
aging rate. Proc Natl Acad Sci U S A.

[153] Schachter F (1998). Causes, effects, and constraints in the genetics of human
longevity. Am J Hum Genet.

[154] Perls T (2004). Dementia-free centenarians. Exp Gerontol.

[155] Blacker D, Albert MS, Bassett SS, Go RC, Harrell LE, Folstein MF (1994). Reliability and validity of NINCDS-ADRDA criteria for
Alzheimer’s disease. The National Institute of Mental Health Genetics
Initiative. Arch Neurol.

